# Recherches Cliniques et Thérapeutiques sur l'Épilepsie, l'Hystérie, et l'Idiotie, compte rendu du service des enfants idiots, épileptiques et arriérés de Bicêtre pendant l'année, 1890

**Published:** 1893-03

**Authors:** 


*Recherches Cliniques et Thérapeutiques sur l'Épilepsie, l'Hystérie, et l'Idiotie, compte rendu du service des enfants idiots, épileptiques et arriérés de Bicêtre pendant l'année,* 1890. Par BOURNEVILLE. Volume XI. Pp. 230. Paris: VVE. BABÉ ET CIE. 1891.

The first part of this book is taken up with an account of the year's work in that part of the great Bicêtre Hospital—namely, the portion which forms the large idiot asylum—which is under the charge of Dr. Bourneville. No name is more honourably associated with the great advance made during recent years in the training of these unfortunates than that of Dr. Bourneville, and those who, like ourselves, have had the privilege of going over that asylum under his courteous guidance could not fail to be struck with its admirable general arrangements, and with the results attained by patient and ingenious methods of training from such unpromising material. Dr. Bourneville is also widely known as one who has contributed largely to the scientific study of idiocy, both in its clinical and pathological aspects. The second part of the work is chiefly devoted to a study of the symptoms and pathological appearances met with in cases of microcephaly, and is illustrated by many excellent plates. The third part contains communications made to the International Medical Congress of Mental Medicine of 1889 by the author on the anatomico-pathological classification of idiocy, on the study of microcephaly and porencephaly, and on sporadic cretinism.

